# Ghrelin mitigates partial body irradiation-induced gastrointestinal acute radiation syndrome by promoting intestinal stem cell regeneration

**DOI:** 10.1186/s10020-025-01399-9

**Published:** 2025-12-24

**Authors:** Satoshi Yamaga, Atsushi Murao, Wayne Chaung, Dmitriy Lapin, Yongchan Lee, Ping Wang, Max Brenner

**Affiliations:** 1 Center for Immunology and Inflammation, The Feinstein Institutes for Medical Research, 350 Community Dr., Manhasset, NY 11030 USA; 2 Departments of Surgery and Molecular Medicine, Zucker School of Medicine at Hofstra/Northwell, Manhasset, NY 11030 USA

**Keywords:** Ghrelin, Ionizing radiation, Intestinal stem cell

## Abstract

**Background:**

Gastrointestinal acute radiation syndrome (GI-ARS) is characterized by disruption of the intestinal barrier function, leading to bacterial translocation and sepsis. Intestinal stem cells are highly radiosensitive and dramatically reduced after radiation injury. Clusterin (Clu)-positive revival stem cells contribute to the restoration of intestinal stem cells. Ghrelin, a gastric peptide hormone, has been shown to improve intestinal integrity in models of inflammatory enteropathy. In this study, we investigated the effects of ghrelin on intestinal stem cell recovery and its potential to mitigate radiation-induced intestinal injury.

**Methods:**

Mice were subjected to 12 Gy partial body irradiation (PBI). Ghrelin at the doses of 2 to 6 nmol per mouse was administered daily for 4 consecutive days, starting at 24 h post-PBI, and survival was monitored for 30 days. To assess intestinal histology, cell proliferation, and intestinal stem cell markers, mice were treated with 6 nmol of ghrelin on days 1, 2, and 3 post-PBI, and on day 4 jejunal samples were collected for qPCR, immunofluorescence, and microcolony assays. Intestinal permeability was assessed in vivo by the leakage of gavage-fed 4-kDa FITC-dextran into the circulation.

**Results:**

Ghrelin administration significantly improved 30-day survival rate following 12-Gy PBI in a dose-dependent manner. Treatment with ghrelin restored villus length and enhanced intestinal barrier integrity. Ghrelin also significantly increased the expression of proliferation markers in the jejunum. Microcolony assays revealed that ghrelin reversed the decrease in BrdU-positive cells following PBI. The mRNA and protein expression of intestinal stem cell markers was decreased after PBI but was restored by ghrelin treatment. Finally, ghrelin significantly increased the population of Clu^+^ population following irradiation.

**Conclusions:**

These findings indicate that ghrelin mitigates radiation-induced intestinal injury by promoting the expansion of Clu^+^ revival stem cells and the recovery of intestinal stem cells. This study highlights the therapeutic potential and identifies the mechanism of action of ghrelin as a medical countermeasure against GI-ARS.

## Background

There is a growing concern about the risk of radiation exposure resulting from detonation of a nuclear weapon or radioactive bomb due to warfare and terrorism (Maiello and Mandel-Ricci 2024). In addition, accidents at nuclear power plants caused by incidents and natural disasters can also lead to radiation exposure (Tanigawa 2021; Hasegawa et al. 2015). Acute radiation syndrome is a clinical syndrome, characterized by hematopoietic, gastrointestinal, neurovascular, and cutaneous subsyndromes (Dainiak and Albanese 2022). Gastrointestinal acute radiation syndrome (GI-ARS) is characterized by breakdown of mucosal barrier, bacterial translocation, and sepsis, resulting in high mortality (Winters et al. 2024). However, there is no medical countermeasure approved by United States Food and Drug Administration (FDA) to mitigate radiation exposure victims with GI-ARS (Winters et al. 2024).

Under homeostatic conditions, the turnover of the intestinal epithelium is maintained by intestinal stem cells, known for their high expression of leucine-rich repeat-containing G-protein coupled receptor 5 (Lgr5) (Barker et al. 2007). Lgr5⁺ intestinal stem cells are capable of self-renewal and generate differentiated progeny that originate the various cell types that compose the intestinal epithelium (Morral et al. 2023). These stem cells are essential for regeneration of the intestinal epithelium following radiation injury (Metcalfe et al. 2014). However, due to their high proliferative activity, Lgr5⁺ intestinal stem cells are highly susceptible to ionizing radiation and are markedly decreased upon radiological exposure (Kim et al. 2017). Recent studies have demonstrated that the progeny of Lgr5^+^ intestinal stem cells can undergo dedifferentiation and reconstitute the intestinal stem cells after radiation injury (Ayyaz et al. 2019; Murata et al. 2020). Furthermore, another study has shown that a transiently expanding population termed as revival stem cells, characterized by the expression of clusterin (Clu), emerges after radiation-induced intestinal injury (Kojima et al. 1999). These Clu⁺ revival stem cells contribute to the restoration of Lgr5⁺ intestinal stem cells and promote regeneration of the intestinal epithelium (Kojima et al. 1999).

Ghrelin, a 28-amino acid peptide, was originally identified as the endogenous ligand of growth hormone secretagogue receptor type 1a (GHSR1a), a receptor that stimulates the pituitary release of growth hormone (Kojima et al. 1999). It exerts a wide range of physiological effects, including regulation of feeding behavior and energy metabolism (Yanagi et al. 2018). Ghrelin has been shown to preserve intestinal integrity in models of sepsis and other inflammatory diseases (Mathur et al. 2020). Moreover, we have identified that ghrelin attenuates intestinal injury in rodent models involving total body irradiation (TBI) (Wang et al. 2015; Shah et al. 2009). However, the effect of ghrelin on intestinal stem cells remains largely unknown. Furthermore, the enteroprotective effects of ghrelin have never been studied in the context of partial body irradiation (PBI), which preserves some of the bone marrow function. Therefore, we hypothesize that ghrelin mitigates GI-ARS via regeneration of intestinal stem cells to improve the prognosis after exposure to high doses of ionizing irradiation.

## Methods

### Experimental animals

Adult C57BL/6 wild-type mice (8 to 12 weeks) were purchased from The Jackson Laboratory (Bar Harbor, ME). Mice were housed in a temperature-controlled environment under 12-h light cycle with ad libitum access to standard rodent chow and water. All animal experiments were performed in accordance with the guidelines for using experimental animals by the National Institutes of Health. All study procedures were approved by the Institutional Animal Care and Use Committee of the Feinstein Institutes for Medical Research (protocol no. 24–1093).

### Partial body irradiation (PBI), ghrelin administration and survival study

Unanesthetized mice were exposed to a single dose of 12-Gy PBI at a dose rate of approximately 1 Gy/min (320 kV, 12.5 mA) with an X-ray irradiation system, X-Rad320 (Precision X-Ray Inc., Madison, CT) as previously described (Chaung et al. 2023). Mice were restrained and placed in a fitted container during irradiation. The hind extremities (fibula, tibia, and feet) were covered with lead shielding, protecting approximately 5% of the bone marrow. Following irradiation, mice were returned to their cages. For survival study, mice were randomly assigned to either the ghrelin treatment group or the vehicle group. Mice received daily subcutaneous injections of human ghrelin (Phoenix Pharmaceuticals, Burlingame, CA) at doses of 2, 4, 6 nmol per mouse daily in the treatment group or vehicle (saline) for 4 consecutive days starting at 24 h after PBI. Mice were monitored daily for 30 days. For intestinal assessment, mice received 6 nmol of human ghrelin or saline daily for 3 consecutive days, starting at 24 h post-PBI. Mice were euthanized on day 4 post-irradiation via CO₂ inhalation followed by cervical dislocation.

### Reverse transcription-quantitative PCR

Segments of the jejunum were harvested, washed three times with cold PBS, snap-frozen in liquid nitrogen, and stored at −80 °C. Total RNA was extracted using TRIzol reagent (Invitrogen, Waltham, MA) according to the manufacturer’s instructions. Complementary DNA (cDNA) was synthesized using reverse transcriptase (Invitrogen) using a Veriti 96-Well Thermal Cycler (Applied Biosystems, Foster City, CA). Quantitative PCR was performed using SYBR Green Master Mix (Applied Biosystems) in a final volume of 20 µL containing forward and reverse primers (final concentration 0.06 mM each), and cDNA. Amplification and analysis were conducted using a StepOnePlus Real-Time system (Applied Biosystems). Mouse β-actin mRNA served as a reference gene for normalization. Relative gene expression levels were calculated using the comparative 2^−ΔΔCT^ method and expressed as fold change relative to the control group. Primer sequences used were as follows: Clu forward (F), 5'-CAGCTGGCTAACCTCACACA-3' and reverse (R), 5'-CTATCTCATTCCGCACGGCT-3’; Mki67 F, 5’-ATCATTGACCGCTCCTTTAGGT-3’ and R, 5’-GCTCGCCTTGATGGTTCCT-3’; Lgr5 F, 5’-CACCTCCTACCTGGACCTCA-3' and R, 5'-GTGTCAAAGCATTTCCAGCA-3'; Olfm4 F, 5’-CAATGTCCTTAGCATTCGCCG-3’ and R, 5’-CCATGACTACAGCTTCCAGGAG-3’; Pcna F, 5’-GAACCTCACCAGCATGTCCA-3’ and R, 5’-AATTCACCCGACGGCATCTT-3’; β-actin F, 5’-CGTGAAAAGATGACCCAGATCA-3’ and R, 5’-TGGTACGACCAGAGGCATACAG-3’.

### Immunofluorescence, immunohistochemistry, and H&E staining

Jejunal tissue was collected and fixed in 10% formalin. Tissue was then paraffin-embedded and sectioned into 5 µm slices, and either mounted unstained or stained with hematoxylin and eosin (H&E) by AML Laboratories (Augustine, FL). For immunofluorescence, paraffin-embedded sections were deparaffinized in xylene and rehydrated in graded ethanol dilutions. The slides were then subjected to antigen retrieval in citrate buffer (Vector Laboratories, Newark, CA) for 15 min at 95 °C. Sections were blocked and permeabilized with PBS containing 10% normal goat serum and 0.2% Triton X-100 for 1 h at room temperature. The following primary antibodies were applied overnight at 4 °C: rabbit anti-Lgr5 (1:200, MA5-32,108, Invitrogen), rabbit anti-clusterin (1:200, 23159S, Cell Signaling Technology, Danvers, MA), and rat anti-Ki67 (1:100, 14–5698-82, Invitrogen). Secondary antibodies included donkey anti-rabbit Alexa Fluor 488 (1:500, A21206, Invitrogen) and goat anti-rat Alexa Fluor 568 (1:500, A11077, Invitrogen), incubated for 1 h at room temperature. Slides were counterstained with DAPI, mounted with ProLong Gold (Invitrogen), and imaged using a Zeiss LSM confocal microscope. Multi-channel Z-stack fluorescence images were obtained using Airyscan SR-4Y fast acquisition mode and processed using FIJI ImageJ (Schindelin et al. 2012). Briefly, extended focus image was generated by performing maximum intensity projection of Z-stacks. Quantitative image analysis was performed by segmentation and measurement processes, which include thresholding, binary layer, and analyzing particles (Lee et al. 2021). Nuclear image was used to count cell number, which was used to normalize the quantification of fluorescence intensity measured in each microscopic field. For BrdU experiments, mice were injected intraperitoneally with 120 mg/kg 5-Bromo-2′-deoxyuridine (BrdU; B5002, Sigma-Aldrich, St Louis, MO) and 12 mg/kg of floxuridine (1271008, Sigma-Aldrich) 2 h before euthanasia to label S-phase cells, and jejunal tissue samples were processed as described above. After deparaffinization, antigen retrieval was performed, followed by 1 N HCl treatment for 10 min on ice and 2 N HCl for 10 min at room temperature. Endogenous peroxidase was quenched with Bloxall Endogenous Blocking Solution (SP-6000, Vector Laboratories) for 20 min at room temperature. Sections were permeabilized in PBS with 0.2% Triton X-100 for 10 min at room temperature, blocked in 2.5% normal horse serum (PK-7200, Vector Laboratories) for 1 h at room temperature, and incubated with mouse anti-BrdU (1:200; 5292S, Cell Signaling Technology) as a primary antibody in PBS with 1% normal horse serum (Vectastain Elite ABC Kit, PK-7200, Vector Laboratories) overnight at 4 °C. After incubation with biotinylated secondary antibody and avidin–biotin peroxidase complex (Vectastain Elite ABC Kit), BrdU signal was visualized using DAB Substrate (SK-4100, Vector Laboratories). Sections were counterstained with hematoxylin QS (H-3404, Vector Laboratories), mounted with ProLong Gold (Invitrogen), and imaged by Nikon Eclipse Ti microscope. After H&E staining, images were obtained at 200 × magnification with Nikon Eclipse Ti microscope. Villus length was determined by measuring the distance from the villus-crypt junction to the villus tip using FIJI ImageJ. The average of 20 villi per mouse was analyzed for quantification. Ki67 was quantified as mean fluorescence intensity of nuclei of cells within the crypt layer of jejunal sections, and BrdU was quantified as the percentage of BrdU-positive cells per crypt.

### Assessment of intestinal permeability

Mice were anesthetized with 2% isoflurane and orally gavaged with 0.5 mL of a 22 mg/mL solution of 4-kDa FITC-dextran (FD4; 60,842–46-8, Sigma-Aldrich) in PBS using a 20-gauge feeding needle. Five hours after gavage, whole blood was drawn by cardiac puncture, and the plasma was collected by centrifugation at 3000 g for 5 min at 4 °C. The fluorescence of FD4 was measured using fluorospectrometry (Synergy H1, BioTek, Winooski, VT) with an excitation wavelength of 485 nm and an emission wavelength of 528 nm. To calculate the fold change of each data point, each experimental value was divided by the sham group’s mean value.

### Statistical analysis

All data are presented as mean and SEM. Statistical comparisons between two groups were made using unpaired, two-tailed Student’s *t*-tests. One-way analysis of variance (ANOVA) and Tukey’s multiple comparisons test were used for multiple groups comparison. P values of < 0.05 were considered statistically significant. Survival was compared among the groups using the log-rank test and Bonferroni adjustment was applied as a multiplier to the P value; thus, the α level remained consistent. Data were analyzed using GraphPad Prism graphing and statistical software (version 10.3.1, GraphPad Software, San Diego, CA).

## Results

### Ghrelin improves 30-day survival after PBI

**Fig. 1:**
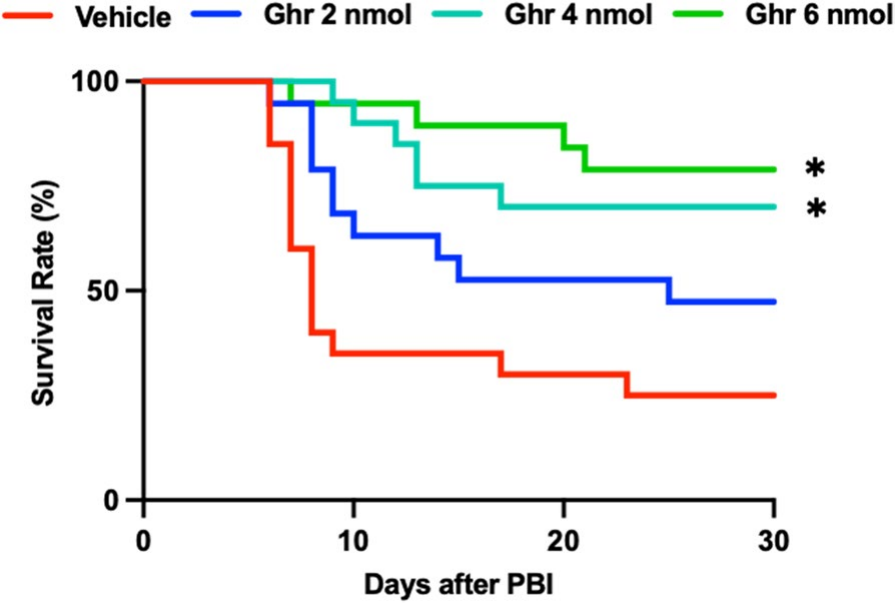
Human ghrelin improves 30-day survival after partial body irradiation (PBI). Mice were exposed to 12-Gy PBI and treated with human ghrelin (2, 4, or 6 nmol per mouse) or vehicle (saline) daily for 4 consecutive days beginning at 24 h post-irradiation. Survival was monitored for 30 days. Groups were compared using the log-rank test (*n* = 20/group). ^*^P < 0.05 vs. vehicle. Ghr, ghrelin; PBI, partial body irradiation

The PBI model is characterized by GI-ARS with severe damage of the intestinal mucosa, a crucial barrier against luminal pathogens and toxins (Winters et al. 2024). Thus, to evaluate the therapeutic potential of ghrelin in severe GI-ARS with partial bone marrow preservation, we subjected mice to 12-Gy PBI and administered human ghrelin at doses of 2, 4, or 6 nmol per mouse daily for 4 consecutive days, beginning at 24 h post-irradiation. Survival was monitored for 30 days. Mice in the vehicle group exhibited a 30-day survival rate of 25%. The survival rate doubled in PBI mice treated with 2 nmol human ghrelin, and further improved in mice treated with 4 and 6 nmol human ghrelin in a dose-dependent manner, reaching 78% in the group receiving 6 nmol of ghrelin (Fig. 1). These results demonstrate that human ghrelin significantly improves the survival of mice exposed to a high dose of radiation with partial bone marrow protection. They also identify 6 nmol as the most effective dose to improve the 30-day survival after PBI.

### Ghrelin mitigates radiation-induced intestinal injury

**Fig. 2:**
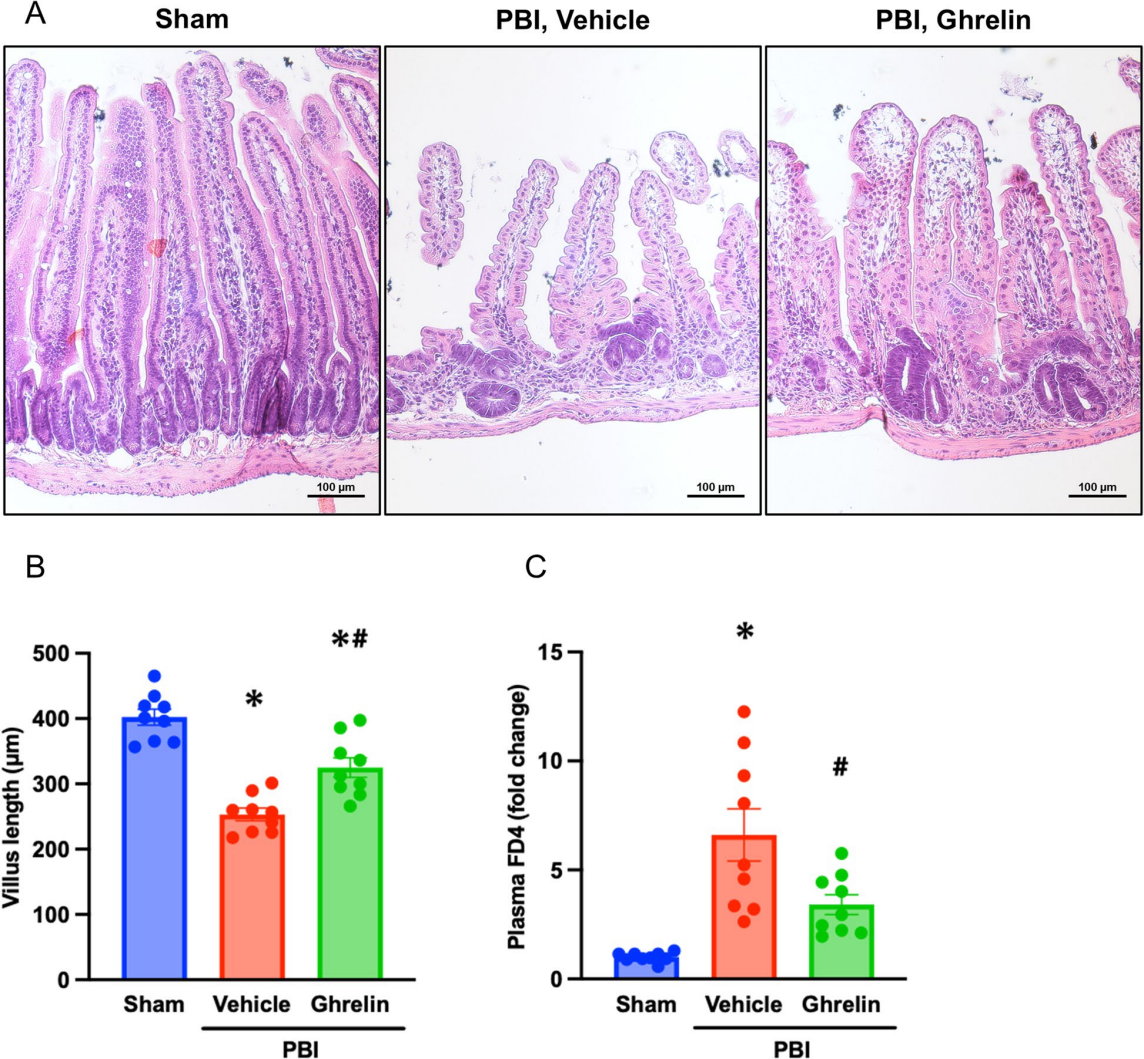
Human ghrelin mitigates radiation-induced intestinal injury. Mice were exposed to 12-Gy PBI and treated with 6 nmol human ghrelin or vehicle (saline) for 3 consecutive days beginning at 24 h post-irradiation. Jejunal samples were harvested on day 4 post-PBI. **A** Representative images of H&E-stained sections. Scale bar, 100 µm. **B** Quantification of villus length. Data are presented as mean ± SEM (*n* = 9/group). **C** Intestinal permeability was assessed by oral gavage with 4 kDa FITC-dextran (FD4) on day 4 post-PBI. Plasma FITC fluorescence was measured 5 h after gavage and expressed as fold change normalized to the sham group. Data are presented as mean ± SEM (*n* = 9/group). Groups were compared using one-way ANOVA and Tukey’s multiple comparison test. ^*^P < 0.05 vs. sham, ^#^ *P* < 0.05 vs. vehicle. FD4, 4 kDa FITC-dextran; PBI, partial body irradiation

To determine the effect of ghrelin on radiation-induced intestinal injury, we then subjected mice to 12-Gy PBI, treated with the optimal dose of ghrelin (6 nmol per mouse at days 1, 2, and 3 post-irradiation), and assessed the jejunal morphology by histological analysis of H&E-stained sections collected at day 4 after PBI. Compared to sham, the jejunal villi of mice subjected to PBI were shortened significantly by 37%. In contrast, ghrelin-treated PBI mice demonstrated a marked 28% improvement in villus length relative to the vehicle group (Fig. 2A, B). These results indicate that ghrelin attenuates intestinal morphologic changes after PBI. We also evaluated the intestinal barrier function after oral gavage with FITC-dextran. Following PBI, plasma FITC fluorescence was significantly elevated in the vehicle group by 6.6-fold compared to the sham group, indicating increased intestinal permeability. Ghrelin treatment significantly reduced plasma FITC-fluorescence by 51% (Fig. 2C). Thus, ghrelin markedly improved the intestinal barrier function after PBI. Taken together, these findings demonstrate that ghrelin mitigates radiation-induced intestinal injury after PBI.

### Ghrelin promotes the regenerative response in the intestine after PBI

**Fig. 3:**
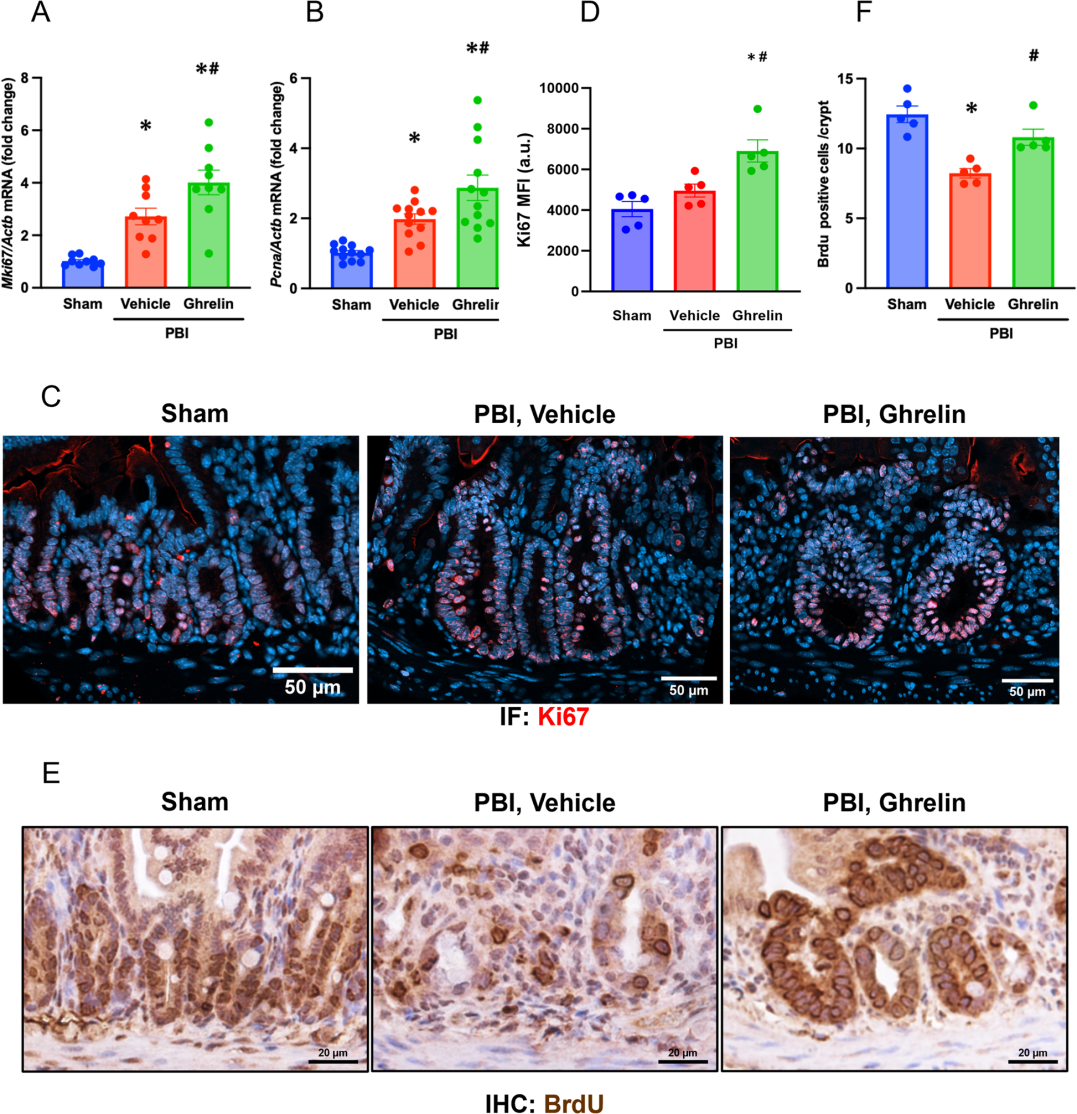
Human ghrelin promotes the regenerative response in the intestine after PBI. Mice were exposed to 12-Gy PBI and administered 6 nmol ghrelin or saline for 3 consecutive days beginning at 24 h post-irradiation. Jejunal samples were collected on day 4 post-PBI. **A, B** mRNA expression levels of *Mki67* and *Pcna* were measured by qPCR. Data are presented as the mean ± SEM (*n* = 9–12/group). **C** Sectioned jejuna were stained by immunofluorescence for Ki67 (red) and nuclear counterstained with DAPI (blue). Representative images; scale bar, 50 µm. **D** Mean fluorescence intensity (a.u., arbitrary units) of Ki67 in the nuclei of cells within the crypt layer of jejunal sections (percentage). Data are presented as the mean ± SEM (*n* = 5/group). **E** In microcolony assay, mice were administered with BrdU 2 h prior to sacrifice and BrdU incorporated into intestinal cross sections was detected (brown). Scale bar, 20 µm. **F** Quantification of BrdU-positive cells per crypt (percentage). Data are presented as mean ± SEM (*n* = 5/group). Groups were compared using one-way ANOVA and Tukey’s multiple comparison test. ^*^P < 0.05 vs. sham, ^#^ *P* < 0.05 vs. vehicle. PBI, partial body irradiation

High doses of radiation collapse the rapid, continuous cell replacement that maintains the integrity and function of the intestinal epithelium monolayer (Winters et al. 2024). Therefore, we next sought to assess the effect of ghrelin on intestinal epithelial cell proliferation after PBI. Ki67 is a nuclear protein associated with cell proliferation, expressed during all active phages of the cell cycle (G1, S, G2, and M), and encoded by the *MKi67* gene (Sun and Kaufman 2018). It has been shown that the intestinal regenerative response is characterized by increase Ki67 expression approximately 4 days after irradiation (Talmasov et al. 2015). We first evaluated *MKi67* mRNA expression in the intestine 4 days after PBI. Compared with the sham group, *MKi67* mRNA expression was significantly elevated by 2.7-fold in the vehicle group, and by 4.0-fold in the ghrelin group (Fig. 3A**)**. We also assessed the gene expression of proliferating cell nuclear antigen (*Pcna*), which is widely recognized as a marker of intestinal epithelial cell proliferation (Sheng et al. 2003). Indeed, a similar upregulation in *Pcna* mRNA expression was observed. *Pcna* expression was significantly elevated by 1.9-fold in the vehicle group, and was further increased in the ghrelin group to 2.9-fold the *Pcna* mRNA level in sham (Fig. 3B). Like the gene expression, at 4 days post-PBI ghrelin treatment showed that mean fluorescence intensity of Ki67 protein in the cell nuclei within the crypt was increased by about 1.4-fold over the level in vehicle (Fig. 3C, D**)**. To further assess epithelial cell proliferation, we performed a microcolony assay in which S-phase cells were labeled with BrdU prior to sacrifice, and then BrdU incorporated into intestinal cross sections was detected (Tustison et al. 2001). Reappearance of regenerating crypts is typically observed within 3.5 to 4 days in this assay (Tustison et al. 2001). Analysis of BrdU-positive cells per intestinal crypt (percentage) at day 4 revealed a significant 33% reduction in the vehicle group, which was improved by 31% in the ghrelin group (Fig. 3E, F). Together, these results indicate that ghrelin treatment enhances intestinal epithelial cell proliferation and supports regeneration following radiation-induced injury.

### Ghrelin facilitates the intestinal stem cell recovery after PBI

**Fig. 4:**
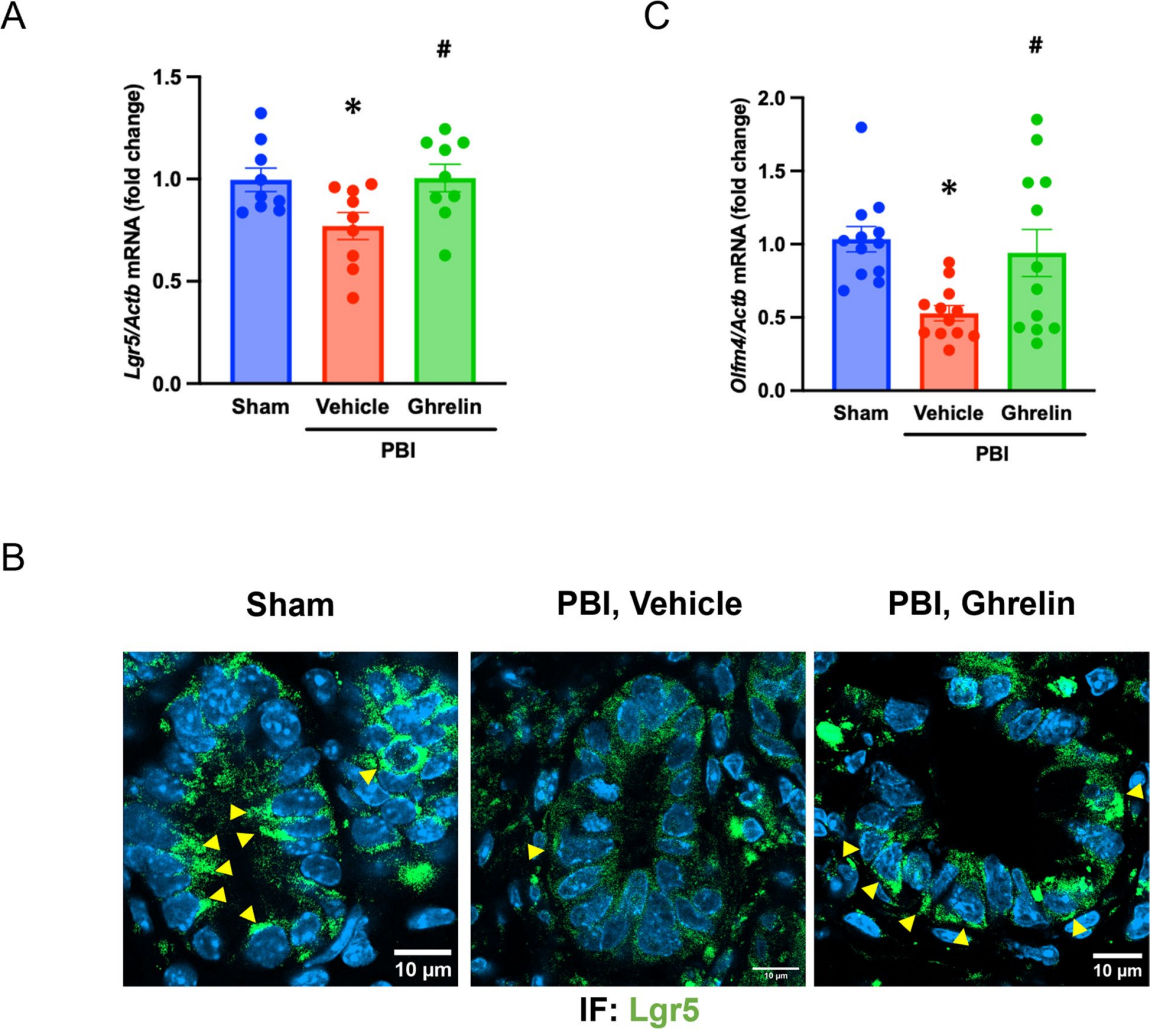
Ghrelin facilitates intestinal stem cell recovery after PBI. Mice were exposed to 12-Gy PBI and treated with 6 nmol ghrelin or vehicle (saline) for 3 consecutive days beginning at 24 h post-irradiation. Jejunal samples were harvested on day 4 post-PBI. **A** *Lgr5* mRNA expression was measured by qPCR. Data are presented as mean ± SEM (*n* = 9/group). **B** Representative immunofluorescence staining for Lgr5 protein (green) and nuclear counterstaining with DAPI (blue). Lgr5 positive cells in the crypt were indicated with yellow arrowhead. Scale bar, 10 µm. Groups were compared using one-way ANOVA and Tukey’s multiple comparison test. ^*^P < 0.05 vs. sham, ^#^ *P* < 0.05 vs. vehicle. PBI, partial body irradiation

We next investigated the effect of ghrelin on the recovery of intestinal stem cells following PBI. Although intestinal stem cells contribute significantly to epithelial regeneration, they are highly susceptible to radiation-induced injury (Kim et al. 2017). The identifying markers for intestinal stem cells include Lgr5 and Olfm4 (Kim et al. 2017). To assess intestinal stem cell recovery, we first examined the gene expression of *Lgr5* and *Olfm4* in the intestine at 4 days post-PBI. *Lgr5* expression levels were significantly downregulated by 33% after irradiation, while ghrelin treatment completely restored *Lgr5* mRNA levels (Fig. 4A). We further evaluated Lgr5^+^ cells in the intestinal crypts by immunofluorescence. Lgr5-positive cells were significantly decreased in the vehicle group following PBI and restored in PBI mice treated with ghrelin (Fig. 4B). Similarly, *Olfm4* expression levels were reduced by 48% in the vehicle group and significantly restored to 94% of sham in ghrelin-treated mice (Fig. 4C). Collectively, these results indicate that human ghrelin promotes the recovery of intestinal stem cells following radiation-induced injury.

### Ghrelin promotes the expansion of the revival stem cell in the intestine after PBI

**Fig. 5:**
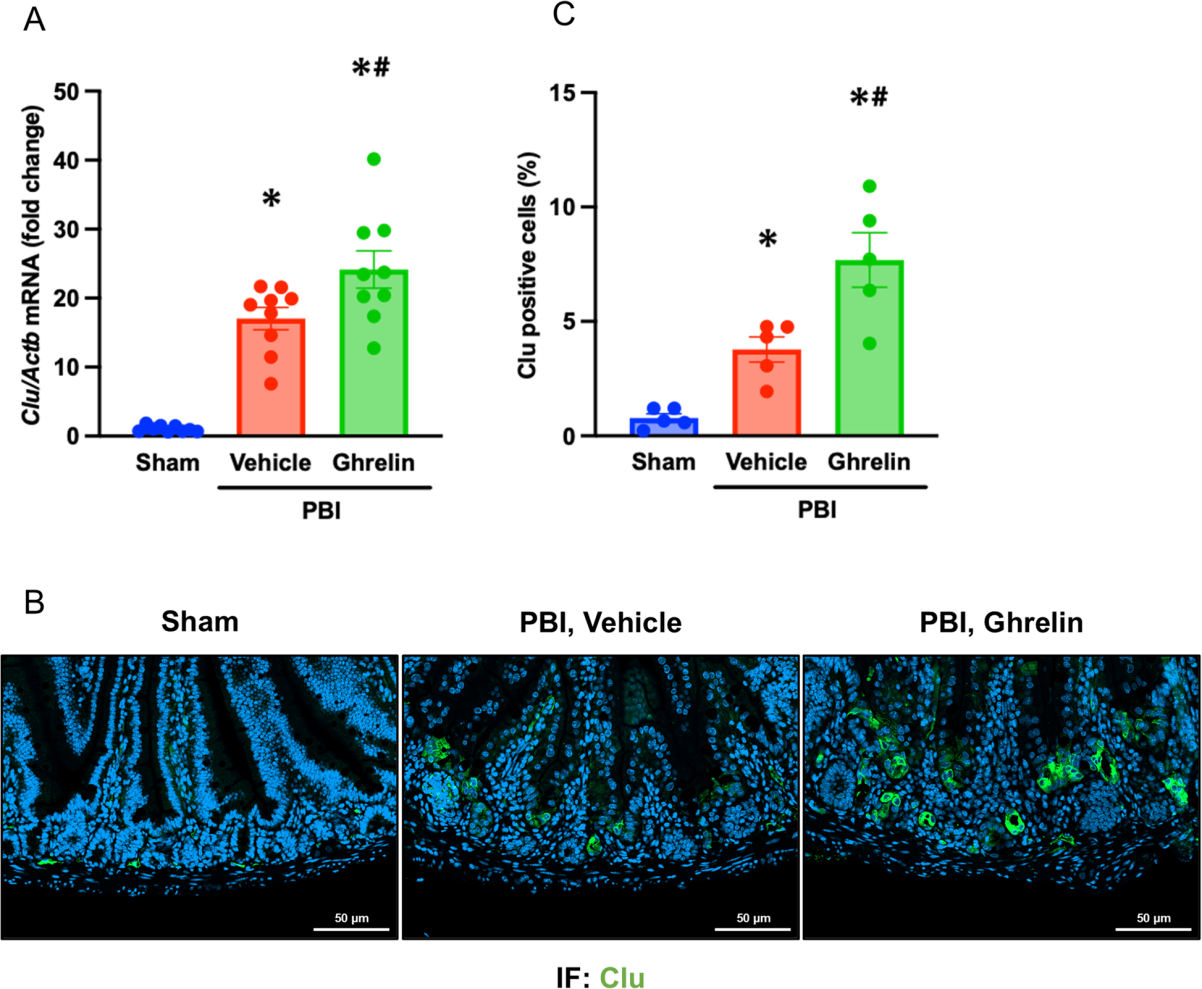
Ghrelin promotes the expansion of revival stem cells in the intestine after PBI. Mice were exposed to 12-Gy PBI and treated with 6 nmol ghrelin or vehicle (saline) for 3 consecutive days, beginning at 24 h post-irradiation. Jejunal samples were collected on day 4 post-PBI. **A** *Clu* mRNA expression was analyzed by qPCR. Data are presented as mean ± SEM (*n* = 9/group). **B** Immunofluorescence staining for Clu (green) and nuclear counterstaining with DAPI (blue). Scale bar, 50 µm. **C** Quantification of Clu-positive cells. Data are presented as mean ± SEM (*n* = 5/group). Groups were compared using one-way ANOVA and Tukey’s multiple comparison test. ^*^P < 0.05 vs. sham, ^#^ *P* < 0.05 vs. vehicle. PBI, partial body irradiation

A recent study has reported that a damage-induced, quiescent stem cell population termed as revival stem cells, characterized by high expression of clusterin (Clu), contributes to reconstitution of Lgr5^+^ intestinal stem cells and promotes intestinal regeneration following injury (Ayyaz et al. 2019). Clu^+^ revival stem cells are rarely observed under homeostatic conditions but undergo transient expansion in response to radiation injury (Ayyaz et al. 2019). To investigate the effect of ghrelin on Clu^+^ revival stem cells, we examined *Clu* gene expression in the intestine at 4 days post-PBI. *Clu* expression levels were significantly upregulated by 17-fold in the vehicle group compared to the sham group, and further increased in mice treated with ghrelin to 24-fold the *Clu* mRNA levels of sham mice (Fig. 5A). Consistent with these changes, immunofluorescence analysis revealed a 4.9-fold increase in the number of Clu^+^ cells in the vehicle group, which was further enhanced to tenfold by ghrelin treatment (Fig. 5B, C). These finding suggest that ghrelin treatment facilitates the expansion of Clu^+^ revival stem cells, thus explaining the associated increase in intestinal stem cells and improved intestinal regeneration in PBI mice treated with ghrelin.

## Discussion

**Fig. 6:**
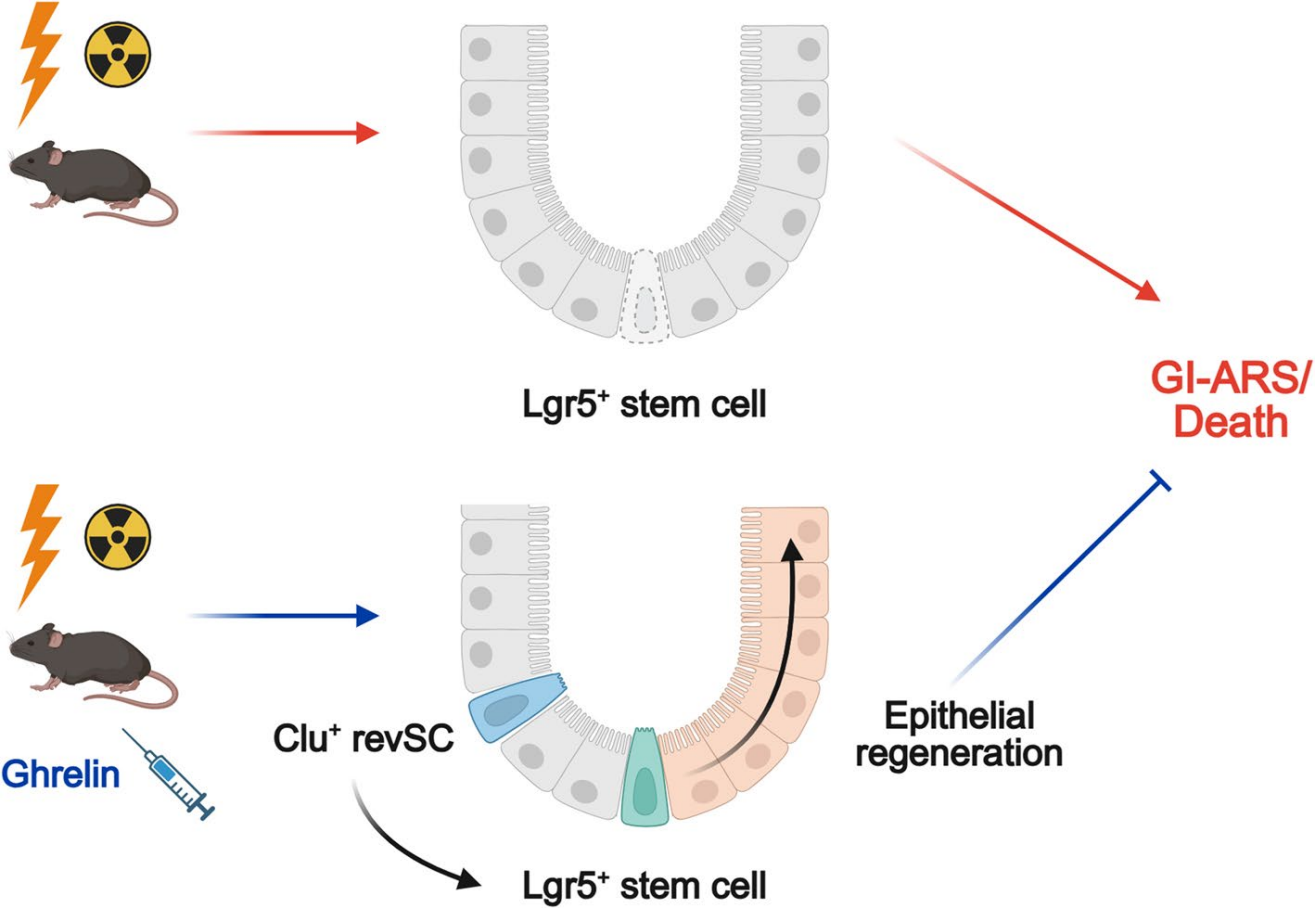
Ghrelin improves radiation-induced intestinal injury. Ghrelin promotes the expansion of Clu^+^ revival stem cells and restoration of Lgr5⁺ intestinal stem cells, facilitating epithelial regeneration and improving survival following PBI. GI-ARS, gastrointestinal acute radiation syndrome; revSCs, revival stem cells. The schema was created in BioRender. Murao, A. https://BioRender.com/cj6md0q

In the present study, we demonstrated that administration of ghrelin improved intestinal mucosal damage, intestinal permeability and 30-day survival in a mouse model of severe PBI. Ghrelin administration promoted proliferative responses within the intestinal crypts. Furthermore, ghrelin treatment enhanced the expansion of Clu^+^ revival stem cells and facilitated the recovery of Lgr5^+^ intestinal stem cells. Collectively, these findings indicate that ghrelin facilitates intestinal healing by promoting post-injury crypt cell plasticity and regeneration, thereby improving outcomes in GI-ARS (Fig. 6).

Ghrelin was first identified as the endogenous ligand for GHSR1a, which was later renamed ghrelin receptor (Kojima et al. 1999). Ghrelin is primarily secreted by the stomach directly into the bloodstream in cycles that increase during fasting and decrease after feeding (Kojima and Kangawa 2005). Ghrelin’s activation of ghrelin receptors in the hypothalamus stimulates appetite (explaining ghrelin’s alternative names orexin and “the hunger hormone”), promotes gastric acid secretion and gastrointestinal motility, and plays a role in regulation of the sleep–wake cycle (Wren et al. 2001; Masuda et al. 2000; Steiger 2007). In addition to these physiological functions, accumulating evidence points to ghrelin’s enteroprotective effect. We and others have shown that ghrelin ameliorates ischemia/reperfusion injury to the gut, sepsis enteropathy, duodenal ulcers, and preclinical models of inflammatory bowel disease (Wu et al. 2008; Li et al. 2019; Ceranowicz et al. 2009; Muthyala et al. 2022). We have also shown that ghrelin has enteroprotective effects against radiation-induced intestinal injury. We have shown that ghrelin attenuates intestinal injury in an RCI model of TBI followed by sepsis and in TBI (Wang et al. 2015; Shah et al. 2009). In the TBI model, ghrelin was shown to reduce epithelial cell apoptosis, preserve the integrity of the intestinal epithelial barrier, and attenuate endotoxemia (Wang et al. 2015). In a combined model of radiation and skin-wound trauma, ghrelin enhanced AKT and ERK activation while suppressing NF-κB and iNOS signaling, thereby reducing inflammatory cytokine production, improving tight junction integrity, and limiting epithelial apoptosis (Kiang et al. 2020). Ghrelin has also been shown to improve bone marrow recovery after radiation exposure (Kiang et al. 2020; 2021; 2018; 2014), which may not only explain ghrelin’s beneficial effects on burns and wound healing in irradiated animals (Kiang et al. 2020; 2014; Liu et al. 2017; 2016), but also on radiation injury to the gut. Therefore, we set out to evaluate human ghrelin’s enteroprotective effect on PBI, a model of severe GI-ARS that preserves some of the bone marrow function in the vehicle and treatment groups (Beach et al. 2023; Fish et al. 2021).

Radiation causes GI-ARS because it disrupts the homeostatic renewal of the intestinal epithelium. The intestinal epithelium is a monolayer containing different cell types that are continuously renewed by Lgr5^+^ intestinal stem cells at the base of the intestinal crypts, migrate to the tip of the villi, and desquamate into the intestinal lumen (Bankaitis et al. 2018). Continuous mitosis renders the fast-cycling Lgr5^+^ intestinal stem cells particularly sensitive to radiation-induced cell death and mitotic catastrophe (Borrego-Soto et al. 2015). Lgr5^+^ intestinal stem cell death leads to collapse of the intestinal epithelium and breakdown of all enterocyte vital functions, including the mucosal barrier against bacteria and luminal contents (Wang et al. 2023; Elliott et al. 2014). As such, restoration of the intestinal stem cell compartment is a critical determinant in the regenerative response after radiation-induced damage and likely represents a key mechanism underlying the beneficial effects of ghrelin on intestinal regeneration. In the present study, we show that ghrelin promotes intestinal integrity, proliferation, and microcolony formation, suggesting a beneficial effect on Lgr5^+^ intestinal stem cells. Indeed, we demonstrated that human ghrelin promotes the regeneration of intestinal Lgr5^+^ intestinal stem cells in mice subjected to TBI.

Having shown that human ghrelin facilitates the recovery of Lgr5^+^ intestinal stem cells following radiation-induced injury, we then considered its mechanism of action. The promotion of intestinal epithelial cell plasticity and dedifferentiation for intestinal regeneration following radiation-induced injury is critically regulated by several signaling pathways, including the Wnt, Notch, and Hippo signaling pathways (Meyer et al. 2022). In a radiation model using human intestinal epithelial cell lines (which express the ghrelin receptor), Kwak et al. demonstrated that ghrelin restored the expression of Notch target genes, whereas the expression of Wnt target genes remained unchanged. Based on these findings, they proposed that ghrelin-mediated recovery of intestinal stem cells following radiation exposure may be primarily dependent on Notch signaling (Kwak et al. 2021). In the present study, we showed that ghrelin administration increased the population of Clu^+^ revival stem cells. These findings raise the possibility that ghrelin promotes the induction of revival stem cell populations within intestinal crypts, thereby contributing to the reconstitution of Lgr5⁺ intestinal stem cells and facilitating intestinal regeneration.

Exactly how human ghrelin facilitates the emergence of Clu⁺ revival stem cells remains unclear. One possibility is that ghrelin directly activates Clu⁺ revival stem cells. However, although intestinal epithelial cells have been shown to express the ghrelin receptor (Englund et al. 2024), its presence in Clu⁺ revival stem cells has not yet been evaluated. Another possibility is that ghrelin activates Clu⁺ revival stem cells indirectly via the vagus nerve connecting the central nervous system to each intestinal crypt (Ten Hove et al. 2021), as suggested by our studies showing that vagotomy abolished ghrelin’s enteroprotection in sepsis enteropathy intestinal ischemia/reperfusion, and RCI models (Shah et al. 2009; Wu et al. 2008; 2009). Therefore, additional studies are needed to elucidate the mechanism by which human ghrelin promotes Lgr5^+^ intestinal stem cell and Clu⁺ revival stem cell recovery following radiation exposure.

The development and use of human ghrelin as a radiomitigator has both advantages and challenges. Human ghrelin has been administered as an infusion or a bolus to over 1850 human subjects participating in more than 100 clinical studies with an excellent safety profile (Garin et al. 2013). Moreover, it can be synthesized in large quantities for mass emergency needs. In healthy humans, ghrelin has a β half-life of 10 min (Akamizu et al. 2008; Tong et al. 2013). While ghrelin’s short half-life in the circulation prevents its accumulation making it safer, it creates posological challenges that are undesired in a mass casualty context and may also limit its enteroprotective effect. This limitation, however, may be circumvented by medicinal chemistry with structural modifications, cyclization, or conjugation, and formulation.

## Conclusions

Human ghrelin confers protective effects against radiation-induced intestinal injury by promoting the regeneration of intestinal stem cells and revival stem cells, thereby restoring the intestinal barrier and improving survival. These findings strongly support the development of human ghrelin as a promising GI-ARS mitigator and provide critical novel insights into its mechanistic of action. These novel insights are directly relevant to the treatment of radiological injuries, and may also be critical to the development of medicines promoting mucosal healing in the contexts of chemotherapy and ischemic, infectious, and autoimmune diseases.

## Data Availability

All the data will be available from the corresponding author upon request.

## Abbreviations

ARS: Acute radiation syndrome
Clu: Clusterin
FD4: 4 KDa FITC-dextran
GI-ARS: Gastrointestinal-ARS
GHSR1a: Growth hormone secretagogue receptor type 1a
FDA: Food and drug administration
PBI: Partial body irradiation
Pcna: Proliferating cell nuclear antigen
revSCs: Revival stem cells
TBI: Total body irradiation
YAP: Yes-associated protein
